# Room-Temperature Superplasticity in an Ultrafine-Grained Magnesium Alloy

**DOI:** 10.1038/s41598-017-02846-2

**Published:** 2017-06-01

**Authors:** Kaveh Edalati, Takahiro Masuda, Makoto Arita, Mitsuaki Furui, Xavier Sauvage, Zenji Horita, Ruslan Z. Valiev

**Affiliations:** 1 WPI, International Institute for Carbon-Neutral Energy Research (WPI-I2CNER), Kyushu University, Fukuoka, 819-0395 Japan; 2 Department of Materials Science and Engineering, Faculty of Engineering, Kyushu University, Fukuoka, 819-0395 Japan; 3 Department of Mechanical Engineering, School of Engineering, Tokyo University of Technology, Hachioji, 192-0982 Japan; 4 Normandie Université, UNIROUEN, INSA Rouen, CNRS, Groupe de Physique des Matériaux, 76000 Rouen, France; 5 Institute of Physics of Advanced Materials, Ufa State Aviation Technical University, Ufa, Russia; 6 Laboratory for Mechanics of Bulk Nanomaterials, Saint Petersburg State University, Saint Petersburg, Russia

## Abstract

Superplasticity, a phenomenon of high tensile elongation in polycrystalline materials, is highly effective in fabrication of complex parts by metal forming without any machining. Superplasticity typically occurs only at elevated homologous temperatures, where thermally-activated deformation mechanisms dominate. Here, we report the first observation of room-temperature superplasticity in a magnesium alloy, which challenges the commonly-held view of the poor room-temperature plasticity of magnesium alloys. An ultrafine-grained magnesium-lithium (Mg-8 wt.%Li) alloy produced by severe plastic deformation demonstrated 440% elongation at room temperature (0.35 *T*_m_) with a strain-rate sensitivity of 0.37. These unique properties were associated with enhanced grain-boundary sliding, which was approximately 60% of the total elongation. This enhancement originates from fast grain-boundary diffusion caused by the Li segregation along the grain boundaries and the formation of Li-rich interphases. This discovery introduces a new approach for controlling the room-temperature superplasticity by engineering grain-boundary composition and diffusion, which is of importance in metal forming technology without heating.

## Introduction

Superplasticity, or ultrahigh tensile elongation prior to failure, is defined as the capacity of a material to be plastically deformed by over 400% under tension^1^. Superplasticity has been of interest for several decades not only from the scientific point of view but also for its high potential in metal forming industry^2^. Superplastic forming has been commercially used to fabricate the complex parts from different Ni-based, Fe-based, Ti-based and Al-based alloys with high dimensional accuracy in a single-cycle process and without any machining^2, 3^. Superplastic forming of Mg-based alloys is currently receiving significant attention because of high strength-to-weight ratio of the alloys and their potential applications in automobile and aerospace industries, but low plasticity of Mg-based alloys at room temperature are still their main drawbacks for structural applications^4^.

Superplasticity occurs only at high homologous temperatures (*T*/*T*_*m*_ > 0.5; *T*: deformation temperature; *T*_*m*_: melting point), where the dominant deformation mechanism changes from the dislocations activity to thermally-activated mechanisms such as grain-boundary sliding^1–3^. This transition in the deformation mechanism causes a significant difference between the plasticity of a material at low and high homologous temperatures. For example, although Mg-based alloys exhibit superplasticity at high temperatures, they show only limited plasticity at room temperature^4, 5^. Therefore, the limited plasticity and deformability of different types of structural materials could potentially be overcome, if the room-temperature deformation mechanism can be modified to grain-boundary sliding to enhance the room-temperature superplasticity.

High-temperature deformation, including superplasticity, at a strain rate of $\dot{\varepsilon }$, is typically defined by the following general stress-creep rate relationship^6^:

1 $$\dot{\varepsilon }=\frac{ADGb}{kT}{(\frac{b}{d})}^{p}{(\frac{\sigma }{G})}^{n}$$

where *A* is a constant (~10), *k* is Boltzmann’s constant, *G* is the shear modulus, *b* is the Burgers vector, $D={D}_{0}\exp (-Q/RT)$ is the diffusion coefficient (*D*_0_: frequency factor; *Q*: activation energy for grain-boundary diffusion; *R*: gas constant), *d* is the average grain size, *σ* is the tensile stress and *p* and *n* are the grain size and stress exponents, respectively. A large number of experimental studies^1, 7^ have shown that, for superplasticity, *D* is the grain-boundary diffusion and *p* is 2. *n* is determined by the strain-rate sensitivity (i.e. the slope of variation of stress versus strain rate in a double-logarithmic plot) using the equation *m* = 1/*n*, in which *m* is 0.5 for pure grain-boundary sliding and decreases with the decreasing contribution of grain-boundary sliding.

Equation (1) suggests that one practical approach to achieving superplasticity at lower temperatures is to refine the grain and reduce the grain size. Although the grain refinement techniques such as severe plastic deformation (SPD) has been widely used in recent decades to reduce the grain size to 100–1000 nm and enhance superplasticity^8–11^, a review of the current results (see ref. 11) shows that superplasticity could be achieved only at homologous temperatures higher than 0.5 *T*_*m*_ such as 923 K for Ti-based alloys, 523 K for Al-based alloys and 473 K for Mg-based alloys. The occurrence of room-temperature superplasticity is currently limited only to alloys with fine-grained microstructure and low melting temperature such as Pb-Sn^12^, Pb-Tl^13^, Sn-Bi^14^ and Zn-Al^15^ alloys. Therefore, to achieve the room-temperature superplasticity in alloys with moderate melting temperatures such as Al-based and Mg-based alloys (i.e. at homologous temperatures of 0.30–0.35 *T*_*m*_), new strategies other than grain refinement should be developed.

Equation (1) suggests that low-temperature superplasticity can also be achieved by enhancing grain-boundary diffusion. The diffusion coefficient increases exponentially with increasing the deformation temperature^6^ and such a strong relationship between the diffusion and temperature is the main reason for the occurrence of superplasticity at high homologous temperatures. However, in our view, the enhancement of diffusion at the grain boundaries can be realized without increasing the temperature by engineering the grain-boundary composition and segregation. A few studies on Y_2_O_3_-based ceramics suggested that the grain-boundary segregation play an important role in the superplastic behavior of the ceramics at high temperatures^16, 17^. Moreover, it was shown that the segregation along grain boundaries can control the energy and mobility of grain boundaries^18^. Although the effect of chemical composition on decreasing the lattice diffusion of metallic materials has been studied for many years^19^, there have been few attempts to control the grain-boundary composition and diffusion to achieve room-temperature superplasticity. Recent developments in structuring metals and alloys at the nanoscale level by means of SPD techniques have opened up new routes to engineering the structure and chemical composition of grain boundaries^20, 21^ and realizing fast diffusivity even at room temperature^22, 23^.

In this study, we hypothesized that increasing diffusion at the grain boundaries in an ultrafine-grained Mg-Li alloy via SPD would lead to superplasticity of the alloy at room temperature. The alloy demonstrated for the first time elongation exceeding 400% at a homologous temperature of 0.35 *T*_*m*_, which originated from fast grain-boundary diffusion and formation of Li-rich grain boundaries. This finding not only introduces a new route to overcome the poor room-temperature plasticity of Mg-based alloys, but also can be employed in low-temperature metal forming technology to fabricate the complex parts without using a heating system.

## Experimental Procedures

**Figure 1:**
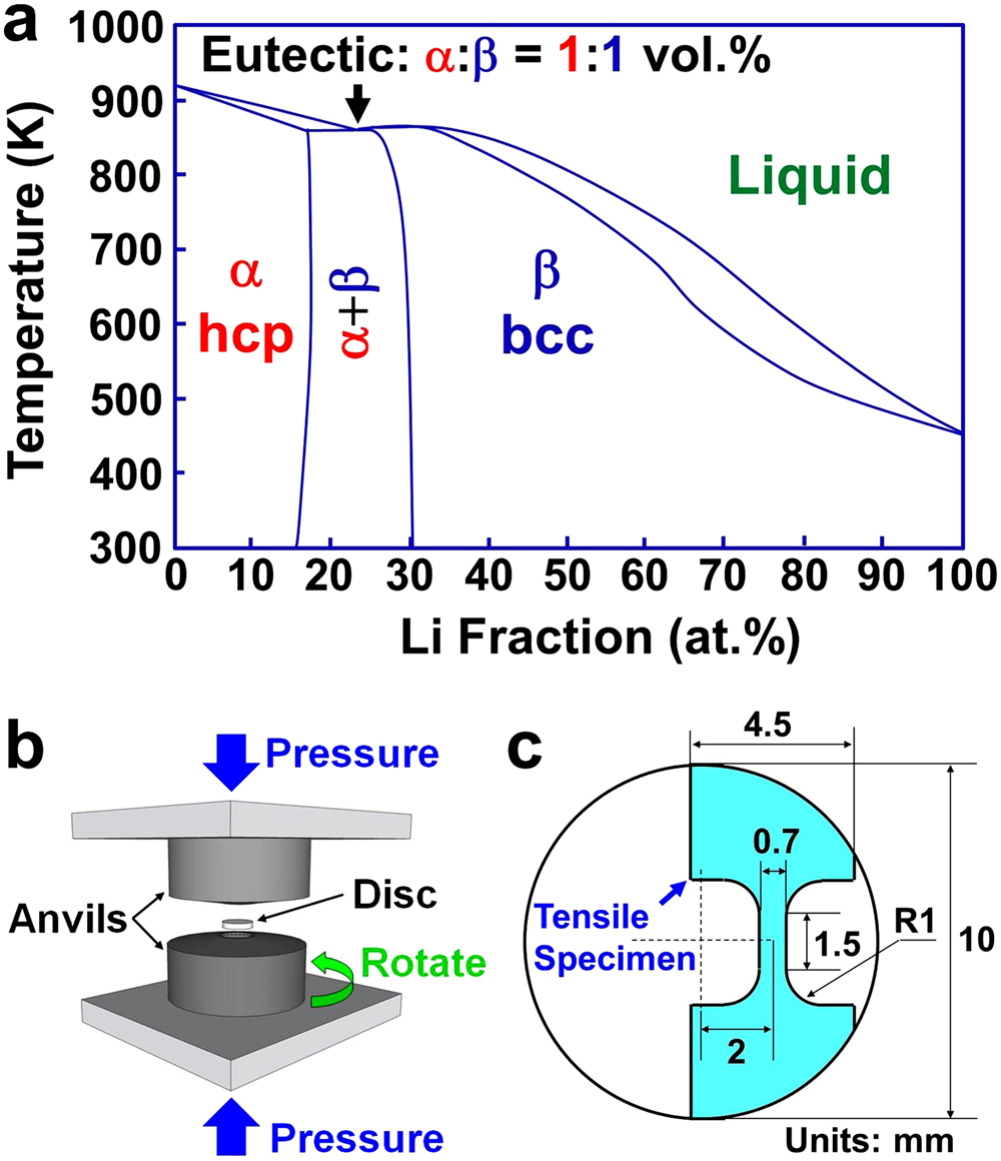
(**a**) Phase diagram of the Mg-Li system. The data were taken from ref. 25 to plot the phase diagram. (**b**) Schematic illustration of high-pressure torsion as an SPD method used in this study. (**c**) The procedure used to make tensile specimens from the discs processed by SPD.

To realize fast grain-boundary diffusion in Mg at room temperature, 8 wt.% (23 at.%) of Li was added to Mg by ingot melting and the as-cast ingot, which was in the form of a rod with a diameter of 25 mm, was extruded at 373 K and its diameter was reduced to 10 mm. Li was selected because it significantly enhances atomic diffusion in the Mg matrix^19, 24^ and improves the superplasticity at temperatures above 473 K^25, 26^ or at 373 K in an ultrafine-grained condition^27^. The alloy had a melting temperature of 861 K containing 50 vol.% of the Mg-rich α phase hexagonal close packed (hcp) structure and 50 vol.% of the Li-rich β phase with body centered cubic (bcc) structure (see the phase diagram of the Mg-Li system in Fig. 1(a))^28^. The concentration of Li was carefully chosen because this amount of Li creates a two-phase eutectic alloy with the highest fraction of Li-rich α/β interphase boundaries^28^. Although the α:β volume ratio in the alloy was 1:1, the fraction of α/β interphase boundaries were not high in the initial extruded bar because of large sizes of each phase, which exceeded 10 μm^26, 27^. To mix the α and β phases effectively and maximize the fraction of Li-rich α/β interphase boundaries, the extruded rod was subsequently cut into discs with a diameter of 10 mm and a thickness of 0.8 mm and processed by SPD through the high-pressure torsion (HPT) method (see the principles of the method in Fig. 1(b))^29^. The disc samples were compressed between two HPT anvils, having flat-bottomed holes of 10 mm diameter and 0.25 mm depth at the center, under a pressure of 6 GPa, and plastic strain was imposed by rotating the lower anvil with respect to the upper anvil for either *N* = 5 or 200 cycles with a rotation speed of 1 rpm at room temperature. Since the magnitude of plastic strain is directly proportional to the number of HPT cycles ($\gamma =2\pi rN/h$, *γ*: shear strain, *r*: distance from disc center, *N*: number of HPT turns or cycles, *h*: thickness of disc^29^), it is expected that the mixing of two phases would be better after rotation for 200 cycles when compared to that after 5 cycles, as reported in other two-phase materials such as Al-Cu alloys^23^ and Cu-W composites^30^.

The plasticity and strain-rate sensitivity of the samples after extrusion as well as after 5 and 200 SPD cycles were examined by performing tensile tests at 300 ± 2 K (room temperature in our laboratory in Fukuoka, Japan) under different strain rates (10^−3^–10^−2^ s^−1^) using samples with gauge dimensions of 0.7 × 0.5 × 1.5 mm^3^. The procedure for making the tensile specimens from the 10 mm diameter discs is shown in Fig. 1(c). It should be noted that the current dimensions of tensile specimens are smaller than the standard size of the tensile specimens because of the small size of the discs processed by SPD.

The structures and microstructures of all samples before and after tensile test were examined at 2–2.5 mm away from the disc center using (i) X-ray diffraction (XRD) analysis using the Cu Kα radiation; (ii) optical microscopy; (iii) atomic force microscopy (AFM) equipped with a NanoScope Analysis software for surface roughness measurement; (iv) scanning electron microscopy in the electron backscatter diffraction mode (SEM-EBSD) using an acceleration voltage of 15 kV; (v) transmission electron microscopy in the bright-field (BF), dark-field (DF) and selected-area electron diffraction (SAED) modes using an acceleration voltage of 300 kV; (vi) energy-filtered transmission electron microscopy (EFTEM) using an acceleration voltage of 200 kV and an energy window of 55–61 eV for Li mapping; and (vii) scanning transmission electron microscopy (STEM) using an acceleration voltage of 200 kV in the high-angle annular dark-field (HAADF) and electron energy-loss spectroscopy (EELS) modes using a convergence angle of 46 mrad and detecting angles of 90–370 mrad.

## Results and Discussion

**Figure 2:**
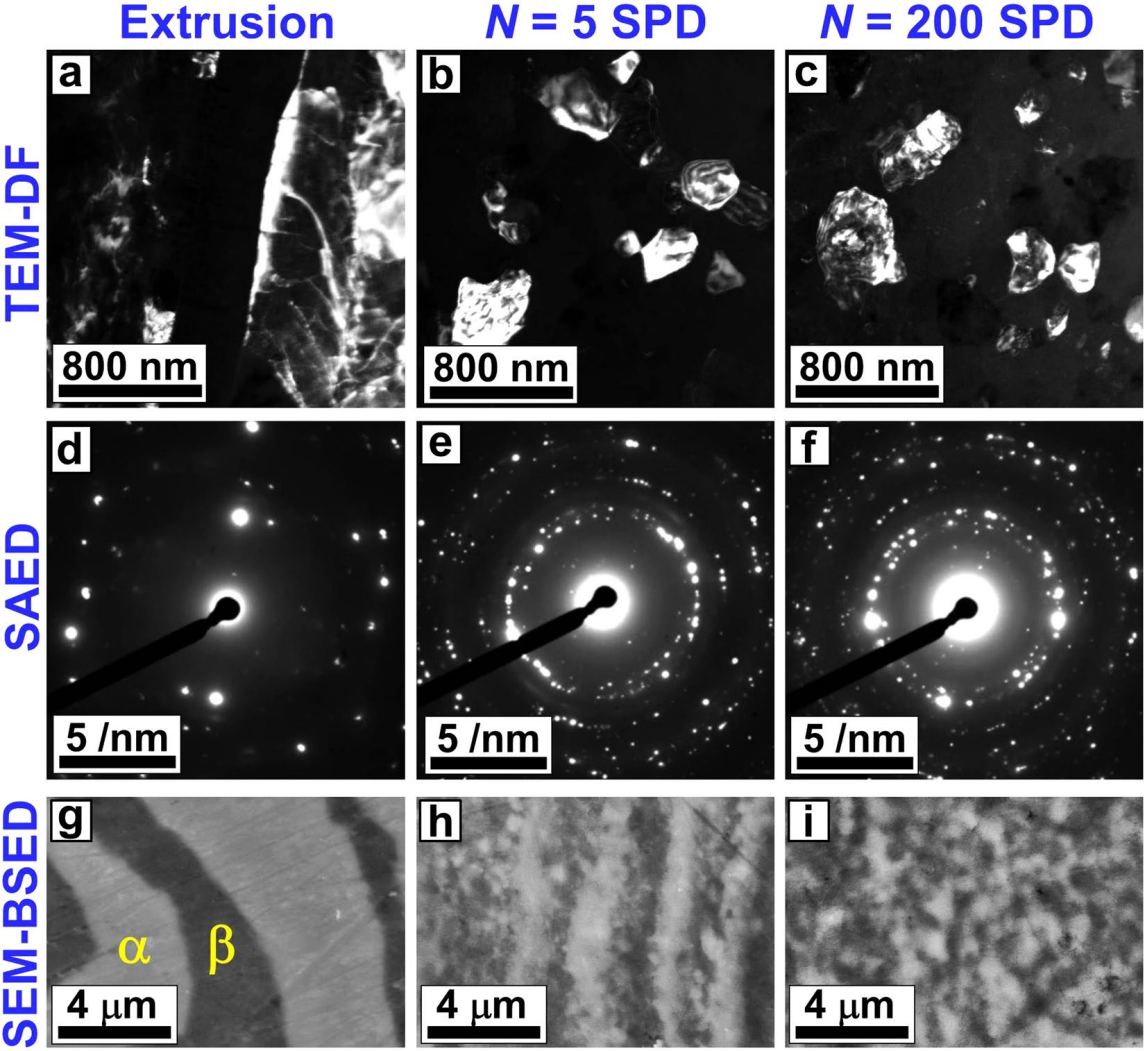
Ultrafine grains with average sizes of 460 nm and 240 nm are formed, and the fraction of α/β interphase boundaries increases to 0.55 μm^2^/μm^3^ and 2.34 μm^2^/μm^3^ in response to SPD processing for *N* = 5 and 200 cycles, respectively. Microstructure of Mg-Li alloy processed with extrusion and SPD for *N* = 5 and 200 cycles. (**a**–**c**) TEM-DF images showing the grain sizes. (**d**–**f**) SAED patterns showing the diffracted beams from the grains. (**a**–**c**) SEM-BSED images showing the distribution of α and β phases.

After extrusion as well as after 5 and 200 SPD cycles, the Mg-Li alloy exhibited a crystal structure containing α phase with a lattice parameter of 0.352 nm and β phases with the lattice parameters of *a* = 320 nm and *c* = 0.514 nm (see XRD profiles in Fig. S1) but with different microstructures. The average size of grains were refined from 2.2 ± 1.4 μm in the initial extruded sample to 460 nm (a bimodal structure with grain sizes in the range of 90–2400 nm) and 240 ± 100 nm after 5 and 200 SPD cycles, respectively, as shown in Fig. 2(a–c). The increase in the number of diffracted beams in the SAED patters after SPD processing, as shown in Fig. 2(d–f), also confirm that the grains were refined after 5 and 200 SPD cycles. Furthermore, as shown in Fig. 2(g–i) and analyzed in details by the linear intercept method^31^, SPD processing induced mixing and fragmentation of the α and β phases and the average phase sizes were reduced from 14.7 μm in the initial extruded sample to 3.6 μm and 0.8 μm after 5 and 200 SPD cycles, respectively. This phase fragmentation results in an increase in the fraction of α/β interface boundaries from 0.16 μm^2^/μm^3^ in the extruded sample to 0.64 μm^2^/μm^3^ and 2.89 μm^2^/μm^3^ after 5 and 200 SPD cycles, respectively. Moreover, the fraction of α/α and β/β grain boundaries increases from 0.45 μm^2^/μm^3^ in the extruded sample to 2.19 μm^2^/μm^3^ and 3.37 μm^2^/μm^3^ after 5 and 200 SPD cycles, respectively (the fractions were calculated per unit volume by considering the grains in the form of hexagons, as shown schematically in Fig. S2 for the sample after 200 SPD cycles). Therefore, despite an increase in the fraction of α/β interface boundaries after 200 SPD cycles (~30% of the total boundaries), the fraction of α/α boundaries is still as large as ~35%.

**Figure 3:**
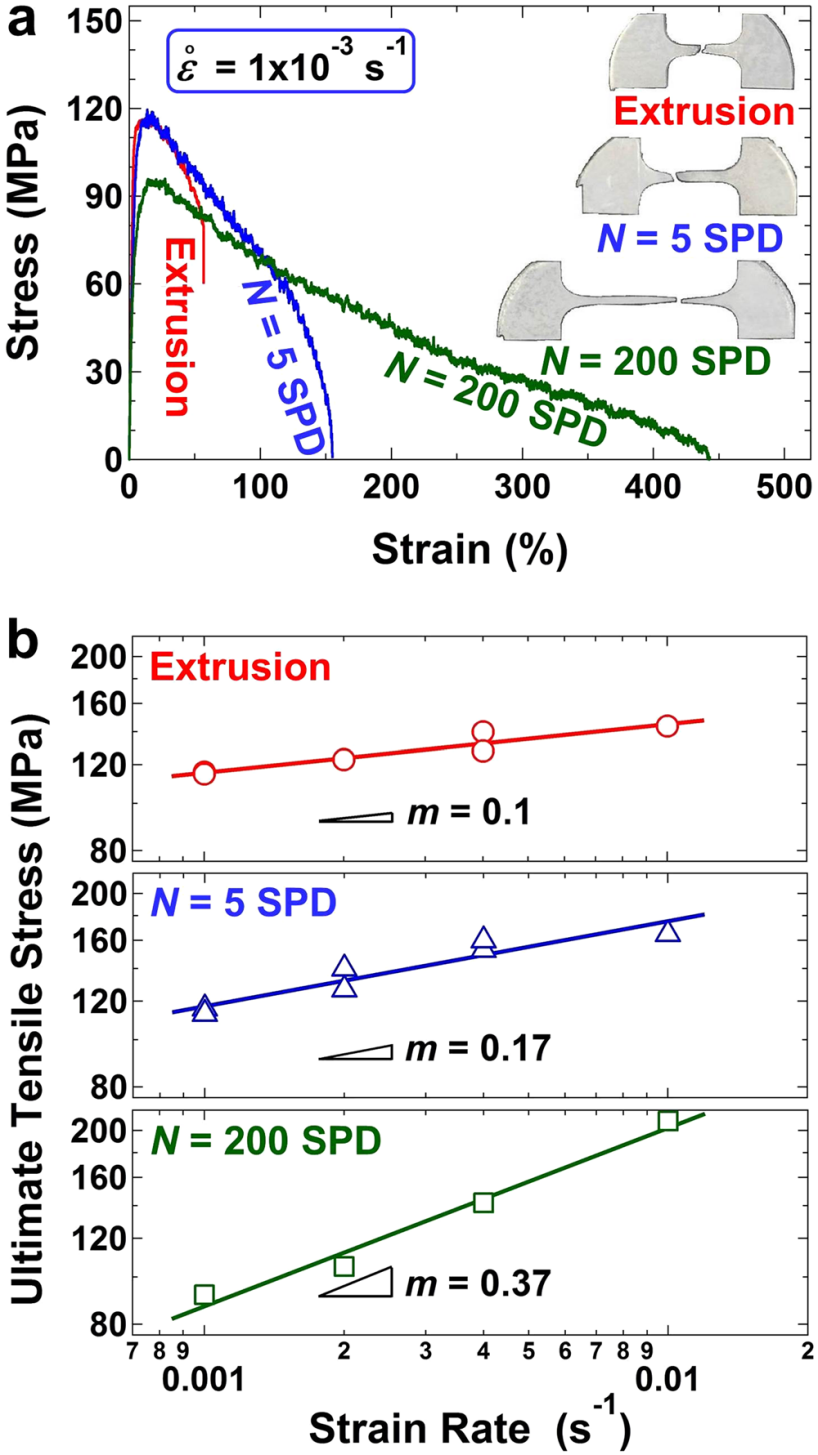
Tensile plasticity and strain-rate sensitivity enhances after SPD processing. Results of tensile tests performed at 300 K for the samples processed by extrusion and SPD for *N* = 5 and 200 cycles. (**a**) Stress-strain curves measured at a strain rate of 1 × 10^−3^ s^−1^. Inset: appearance of tensile specimens after pulling to failure. (**b**) Ultimate tensile stress as a function of strain rate; the slopes represent the strain-rate sensitivity, *m*. The sample after 200 SPD cycles exhibits room-temperature superplasticity with *m* = 0.37.

To determine the plasticity of the three samples, we generated tensile stress-strain curves, obtained at initial strain rates of 10^−3^–10^−2^ s^−1^ and a temperature of 300 K (room temperature in our laboratory in Fukuoka, Japan). Figure 3(a) shows the stress-stress curves obtained at an initial strain rate of 1 × 10^−3^ s^−1^ including the appearance of samples after tensile test. The double-logarithmic plots of Fig. 3(b) show the ultimate tensile stress as a function of the strain rate and the slopes of plots represent the strain-rate sensitivity. Although the extruded sample exhibited limited plasticity with a strain-rate sensitivity of *m* = 0.1, the sample after 5 cycles of SPD showed high plasticity as 160% but not superplasticity. The sample after 200 cycles of SPD exhibited an elongation of 440%, which is well in the accepted range of superplasticity^1^, with a high strain-rate sensitivity of *m* = 0.37. The large elongation at room temperature (0.35 *T*_*m*_) and enhanced strain-rate sensitivity of the specimen processed by 200 SPD cycles suggests that the main deformation mechanism of the sample at room temperature was successfully modified to grain-boundary sliding^1^.

**Figure 4:**
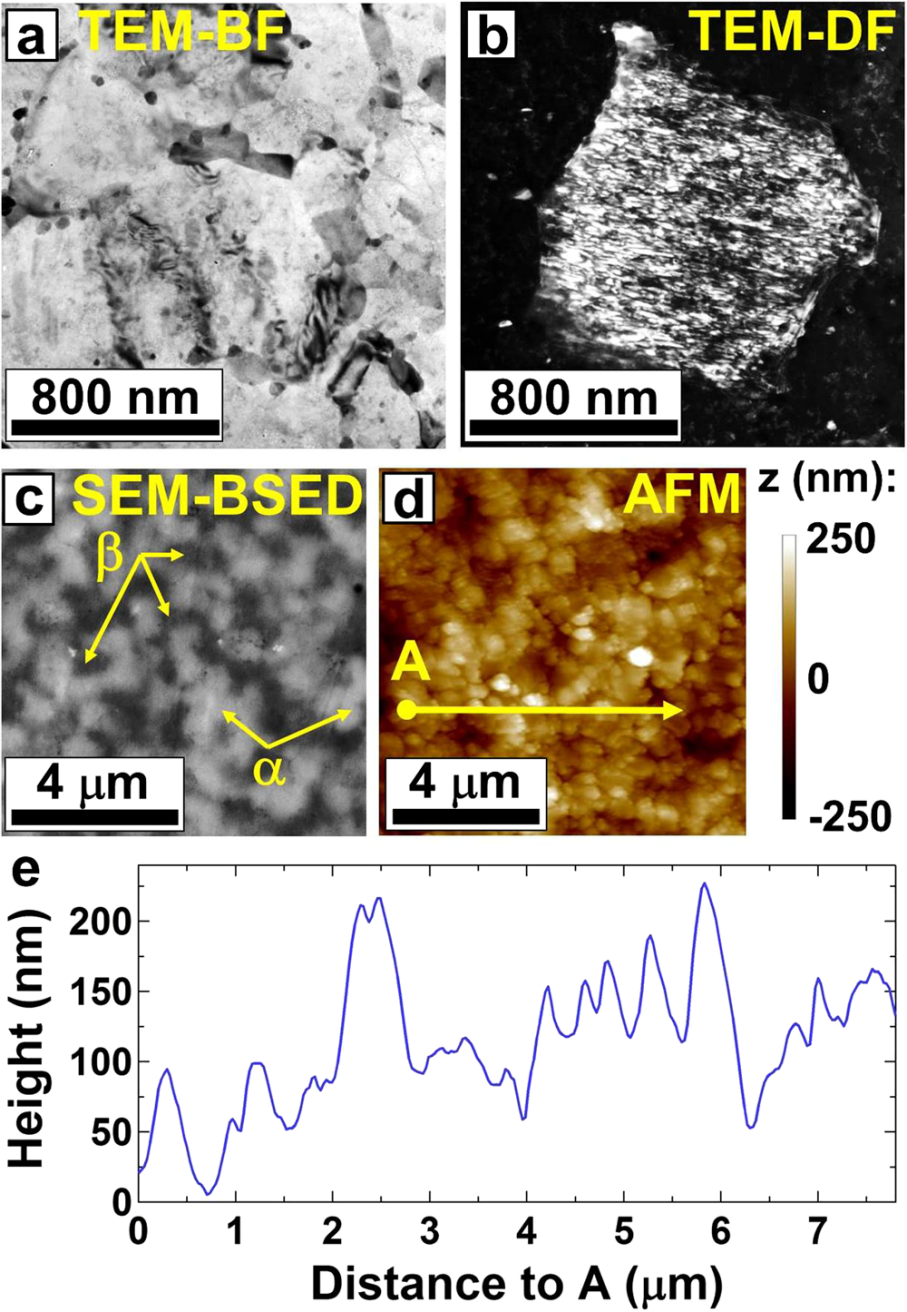
Absence of elongated grains, occurrence of grain coarsening, homogenous distribution of α and β phases and increase in the surface roughness after tensile test indicates the occurrence of grain boundary sliding. Microstructure and surface conditions of the sample processed by *N* = 200 cycles of SPD after tensile test to failure. (**a**) TEM-BF and TEM-DF images showing the morphology of the grains. (**c**) SEM-BSED image showing the distribution of α and β phases. (**d**) AFM image showing the surface roughness. (**e**) Plot of surface morphology along the direction indicated by an arrow in (**d**).

To confirm the successful conversion to grain-boundary sliding in the sample after 200 cycles of SPD, we analyzed the grain size, phase distribution and surface texture of the alloy both before and after the tensile test. After the tensile test, although the elongation was as large as 440%, elongated grains could not be observed in the microstructure, as shown in Fig. 4(a). The microstructure of the sample after tensile test contained grains with an average size of 480 ± 260 nm, which was larger than the average grain size of 240 ± 100 nm prior to the tensile test (see Fig. 2(f)). Such an increase in the grain size after tensile test suggests that a typical strain-induced grain coarsening should have occurred during the superplastic deformation^32^, as shown in Fig. 4(b). Most of the grains were free of dislocations and possessed smooth grain boundaries, as shown in Fig. 4(a), which is in agreement with the typical grain features observed after grain-boundary sliding^1–3^. The distribution of the α and β phases after the tensile test, as shown in Fig. 4(c), was similar to that before the test, as shown in Fig. 2(c). Furthermore, although the surface was polished before the tensile test, evaluation of surface roughness by AFM and NanoScope Analysis software, as shown in Fig. 4(d), confirmed that the surface roughness appears after the tensile test. The submicrometer-level surface-roughness periodic of ~500 nm observed after the tensile test (see the peak-to-peak distances in Fig. 4(e)) was consistent with the average grain size of 480 ± 260 nm measured after the tensile test, confirming that the appearance of surface roughness is consistent with grain boundary sliding. The irregular periodicity of the surface roughness should be due to the presence of two different phases with different types of α/α, α/β and β/β boundaries. Although it is hard to make a direct correlation between the surface roughness in Fig. 4(e) and the atomic or phase structure because of the limitations of the AFM method, a comparison between Fig. 4(c) and (d) suggests that the surface-roughness periodic for the α and β phases is reasonably the same, confirming that the sliding of all boundaries (α/α, β/β and α/β) should contribute to the superplasticity. The surface roughness was more pronounced in the sample after 200 SPD cycles than in the extruded sample, as shown in Fig. S3, which displays the surface conditions of the three samples. The sample after 5 cycles of SPD also shows partial surface roughening in some regions. Taken together, these results indicate the occurrence of significant grain-boundary sliding for the specimen after 200 cycles of SPD and partial grain-boundary sliding for the sample after 5 cycles of SPD.

To estimate the contribution of grain-boundary sliding (*ε*_*GBS*_) to the total elongation (*ε*_*T*_) after 200 cycles of SPD, a tensile specimen was elongated by 90% under a strain rate of 1 × 10^−3^ s^−1^ at a temperature of 300 K. The sample was then polished smoothly, further elongated by 30% and examined by AFM. The contribution of grain-boundary sliding was estimated using the following previously-established equation^2, 3^:

2 $${\varepsilon }_{GBS}=1.4\frac{v}{d}$$

In this equation, *v* is the average step size normal to the surface, which was estimated by comparing the surface conditions before and after the tensile test in the AFM images. Because the average of *v*, measured by AFM over a few regions with a total area of 2 μm^2^, increased by approximately 30 nm after the 30% elongation (some representative results of AFM are shown in Fig. S4), the contribution of grain-boundary sliding to the total elongation was estimated approximately 60%. This confirms that the main deformation mechanism at room temperature was grain-boundary sliding.

**Figure 5:**
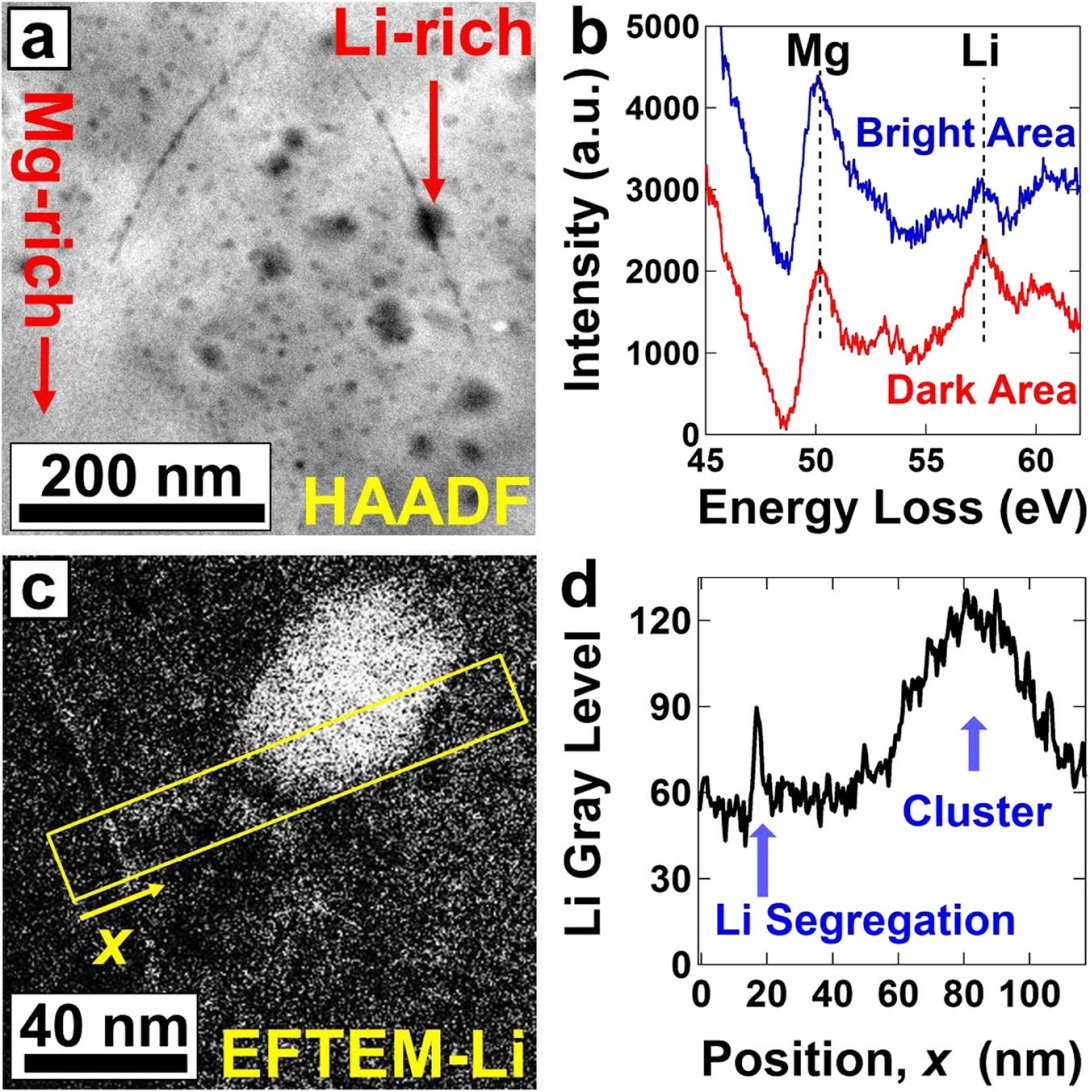
Li segregates along the boundaries in response to SPD processing. Microstructure of the sample processed by *N* = 200 cycles of SPD. (**a**) HAADF image showing the distribution of Mg and Li atoms. Bright and dark contrasts correspond to the Mg-rich and Li-rich regions, respectively. (**b**) EELS spectra obtained from the bright and dark regions, indicated by arrows, in the HAADF image. Comparison between the peaks for Mg and Li shows the distribution of Mg and Li atoms. (**c**) EFTEM mapping for Li in which the dark and bright contrasts correspond to Mg-rich and Li-rich regions, respectively. (**d**) Changes in the gray level along the *x* direction in the EFTEM image. An increasing in the gray level corresponds to an increase in the fraction of Li.

To study the origin of the enhanced grain-boundary sliding and room-temperature superplasticity after 200 cycles of SPD, we carefully inspected the grain boundaries using a Cs-corrected microscope equipped with STEM-HAADF, EELS and EFTEM modes (See Fig. 5). Examination of boundaries using the STEM-HAADF mode, as shown in Fig. 5(a) confirmed the presence of dark contrast in many α/α grain boundaries, which should be due to the presence of Li which has a lower atomic number than Mg. To confirm that that the dark contrasts were not artifacts, EELS spectra were taken from the dark regions and compared with the spectra in the bright regions. As shown in the EELS spectra of Fig. 5(b), the intensity of Li peak is higher in the dark regions than that in the bright regions, confirming that the concentration of Li atoms is high in the dark regions. Moreover, the boundaries were examined with EFTEM using an energy window of 55–61 eV, corresponding to Li. As shown in Fig. 5(c), the bright contrast, which corresponds to the Li-rich regions, appeared in the boundaries. The segregation of Li along grain boundaries is shown more clearly in the EFTEM line profile of Fig. 5(d). Since the grain boundaries in severely-deformed materials have non-equilibrium states similar to the ones in irradiated metals, the driving force for the segregation of solute atoms along the boundaries might be the reduction of the boundary energy, as discussed earlier by theoretical studies^18^ and atomic-scale experimental analyses^33^. Another feature observed in Fig. 5 is the presence of Li-rich, β-type clusters in the α phase, which originate from the extruded sample (see Fig. S5). These clusters, which presented in three examined samples, might not have a direct effect on the occurrence of superplasticity because they are mainly located within the grains interior. Taken together, these results indicate that Li atoms have segregated along the α/α boundaries after 200 cycles of SPD and because α/α interfaces exhibit lower grain-boundary diffusion than β/β and α/β ones^19, 24^, the segregation of Li along the α/α boundaries enhanced grain-boundary diffusion, making grain-boundary sliding and superplasticity more favorable.

Because the data for lattice diffusion in the Mg-Li is already available in the literature^19, 24^ and the activation energy for grain-boundary diffusion *Q* is typically 50% of the activation energy for lattice diffusion *Q*_*L*_ (*Q* = 1/2 *Q*_*L*_)^19^, it is possible to estimate the grain-boundary diffusion in the Mg-Li system at *T* = 300 K ($D={D}_{0}\exp (-0.5{Q}_{L}/RT)$ ^19^) and compare the data with the diffusion coefficient estimated for superplasticity using Equation (1). The grain-boundary diffusion was calculated to range from 1.6 × 10^−16^ m^2^ s^−1^ in the Mg-rich side to 3.7 × 10^−10^ m^2^ s^−1^ in the Li-rich side (*Q*_*L*_ and *D*_*0*_ are 138.2 kJmol^−1^ and 2.4 × 10^−3^ m^2^ s^−1^ in the Mg-rich side and 55.3 kJmol^−1^ and 1.75 × 10^−4^ m^2^ s^−1^ in the Li-rich side, respectively^19^). Using Equation (1) for our experimental data and considering $\dot{\varepsilon }$, *G*, *b*, *T*, *d*, *σ* and *m* as 1 × 10^−3^ s^−1^, 17.3 GPa, 0.320 nm, 300 K, 240 nm, 92 MPa and 0.37, respectively, the grain-boundary diffusion for superplasticity was evaluated to be *D* = 5.8 × 10^−14^ m^2^s^−1^. This value is smaller than the room-temperature diffusivity along the Li-rich β/β and α/β boundaries (3.7 × 10^−10^ m^2^s^−1^), but it is two orders of magnitude higher than the room-temperature diffusivity along the Mg-rich α/α boundaries (1.6 × 10^−16^ m^2^ s^−1^). The β/β and α/β boundaries can exhibit boundary sliding at room temperature because of fast diffusivity along these boundaries^19^, but the α/α boundaries are barriers for uniform superplasticity because of slow diffusivity along the α/α boundaries at room temperature. Since the slowest phenomenon, i.e. diffusivity along the α/α boundaries, is the controlling parameter for superplasticity^1–3^, it is then concluded that the room-temperature diffusivity along the α/α boundaries should be enhanced by two orders of magnitude in this study. This enhanced diffusivity for room-temperature superplasticity in the ultrafine-grained Mg-Li alloy is still lower than the required diffusivity along the α/α boundaries for high-temperature superplasticity (7.2 × 10^−13^ m^2^s^−1^ at 0.5 *T*_m_)^19^ because the grain sizes at high temperatures are usually larger than those at room temperature (Equation (1) suggests that the required diffusivity for superplasticity increases with increasing the grain size). Such an increase in the room-temperature diffusivity is consistent with the enhancement of the Li concentration along the boundaries, as discussed earlier in Fig. 5, suggesting that room-temperature grain-boundary sliding and superplasticity could be achieved by modifying the chemical composition of the grain boundaries (via grain-boundary segregation or by increasing interphase boundaries).

Taken together, the results from our study show that adding Li to Mg and processing the alloy by SPD for refining the grains and controlling the chemistry of grain boundaries increases the rate of grain-boundary diffusion, leading to grain-boundary sliding and superplasticity of the alloy at room temperature. This transition to grain-boundary sliding cannot be attributed only to grain refinement, because the sample after 5 cycles of SPD also contained ultrafine grains but did not exhibit superplasticity. An earlier paper also showed that the sample after 5 cycles of SPD could not exhibit superplasticity below a temperature of 373 K^27^. Moreover, earlier attempts to achieve room-temperature superplasticity either in ultrafine-grained Mg-based alloys^11^ or specifically in Mg-Li alloys^25, 26^ were not successful and the alloys exhibited superplasticity at 473 K or higher temperatures. The main reason for the generation of significant grain-boundary sliding and room-temperature superplasticity in our study was the segregation of Li at the grain boundaries and the increase in the fraction of Li-rich α/β interphase boundaries from 0.13 μm^2^/μm^3^ to 2.34 μm^2^/μm^3^, which enhanced grain-boundary diffusion. Earlier studies also showed that the grain-boundary segregation has a significant influence on the mobility of grain boundaries not only in metallic materials^18^ but also in superplastic ceramics^16, 17^. Because grain-boundary segregation^20, 21^ and enhancement of the fraction of interphase boundaries^23, 30^ can be achieved by SPD processing in different kind of materials, we believe that the engineering chemistry of grain boundaries can be used to enhance the room-temperature superplasticity by the appropriate selection of elemental additives (such as addition of Li, Na, Ca, Sr, Se, Bi and Te to Mg which can enhance the diffusivity)^34^ and SPD processing. The SPD process that was used in this study was HPT method, which can process the disc samples with small sizes, but numerous SPD techniques have been developed in recent decades^9, 35^ which can be employed to process the samples with large sizes for low-temperature forming industries.

## Conclusions

In summary, this study suggests that, while reduction of grain size enables the achievement of superplasticity at high temperatures, the modification of grain-boundary diffusion through engineering of grain-boundary composition is an effective method to enhance grain-boundary sliding and realize room-temperature superplasticity in Mg-based alloys. This approach, which is not necessarily limited to Mg-based alloys, opens up a new path to enhancing the deformability of structural materials at room temperatures, which can be used in metal forming technology to make complex specimens without need to use the heating or machining systems.

## Electronic supplementary material

Supplementary figures
