# Supplementary material for: Room-Temperature Superplasticity in an Ultrafine-Grained Magnesium Alloy
Source: Sci Rep. 2017 Jun 1;7:2662. doi: 10.1038/s41598-017-02846-2 (PMC5453936; doi:10.1038/s41598-017-02846-2)
Supplement: Supplementary file 1 — Supplementary figures [file 41598_2017_2846_MOESM1_ESM.pdf]

Supporting Information

Room-Temperature Superplasticity in an Ultrafine-Grained Magnesium Alloy

Kaveh Edalati[1,2,*], Takahiro Masuda[2], Makoto Arita[2], Mitsuaki Furui[3], Xavier Sauvage[4], Zenji Horita[1,2] and Ruslan Z. Valiev[5,6,*]

[1] WPI, International Institute for Carbon-Neutral Energy Research (WPI-I2CNER), Kyushu University, Fukuoka 819-0395, Japan

[2] Department of Materials Science and Engineering, Faculty of Engineering, Kyushu University, Fukuoka 819-0395, Japan

[3] Department of Mechanical Engineering, School of Engineering, Tokyo University of Technology, Hachioji 192-0982, Japan

[4] Normandie Université, UNIROUEN, INSA Rouen, CNRS, Groupe de Physique des Matériaux, 76000 Rouen, France

[5] Institute of Physics of Advanced Materials, Ufa State Aviation Technical University, Ufa, Russia

[6] Laboratory for Mechanics of Bulk Nanomaterials, Saint Petersburg State University, Saint Petersburg, Russia

***Corresponding authors:**

Kaveh Edalati (E-mail: kaveh.edalati@zaiko6.zaiko.kyushu-u.ac.jp)

Ruslan Z. Valiev (E-mail : ruslan.valiev@ugatu.su)

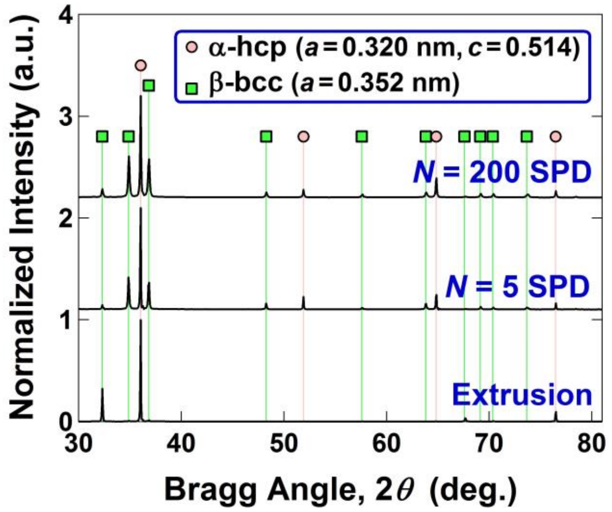

Figure S1. The Mg-Li alloy contains 50 vol.% of α phase with the hcp structure with lattice parameters of a = 0.320 nm and c = 0.514 nm, and 50 vol.% of β phase with the bcc structure with a lattice parameter of a = 0.352 nm. XRD profiles of the Mg-Li alloy processed with extrusion and SPD for N = 5 and 200 cycles. The intensity of profiles was normalized by the intensity of the most intense peak at a Bragg angle of 36° for better visibility.

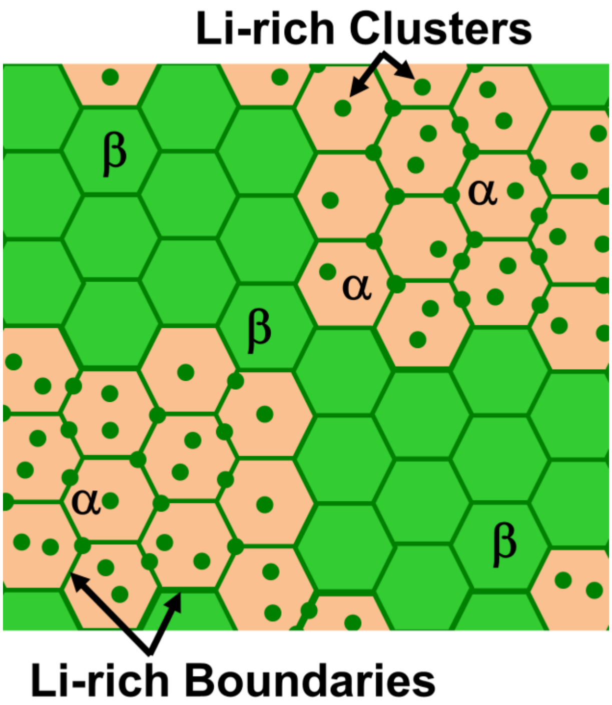

Figure S2. Schematic illustration of ultrafine-grained Mg-Li alloy processed with SPD for *N* = 200 turns.

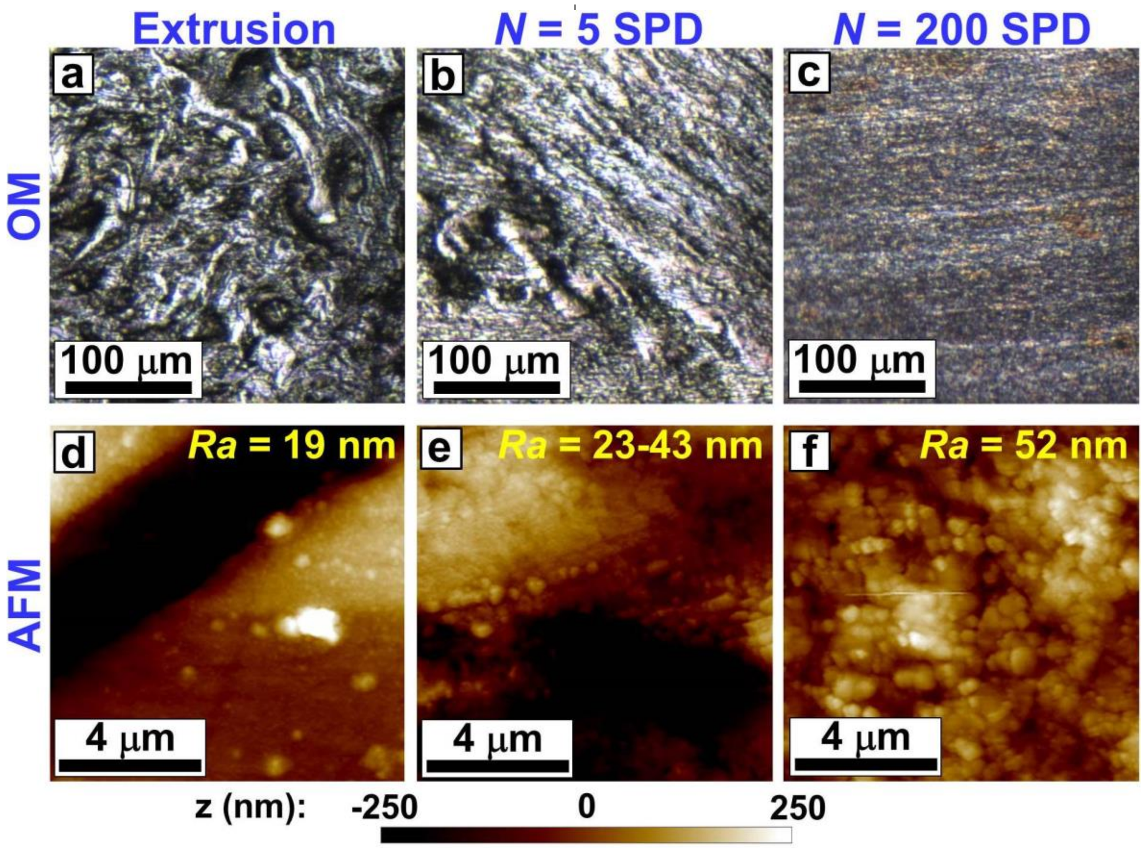

Figure S3. The deformation becomes more uniform and the surface roughness at the sub-micrometer level increases after SPD processing for large number of cycles as N = 200. Surface conditions for the samples processed by extrusion and SPD for N = 5 and 200 cycles after the tensile test to failure. (a–c) OM images showing the uniformity of tensile deformation. (d–f) AFM images showing the surface roughness. *Ra* represents the surface roughness after the tensile tests. Horizontal axis is the tensile direction.

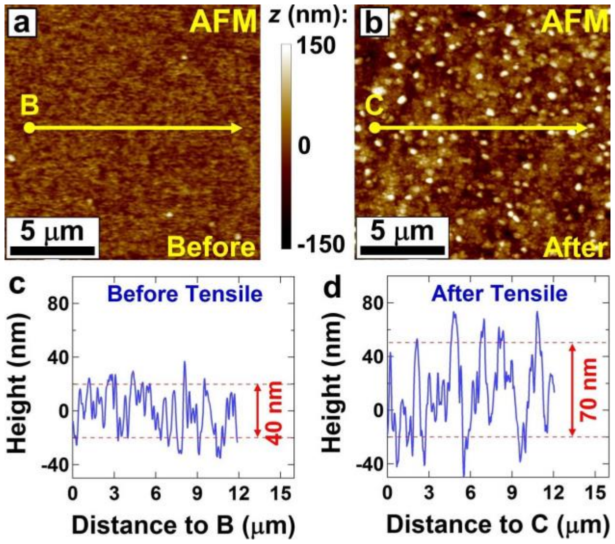

Figure S4. The surface roughness increases, and the average step size normal to the surface increases approximately 30 nm after tensile test for 30% elongation. Surface conditions for the sample processed by SPD for N = 200 cycles before and after the tensile test for 30%. (a, b) AFM images showing the surface roughness. (c, d) Plot of surface morphology along the direction indicated by arrows in AFM images.

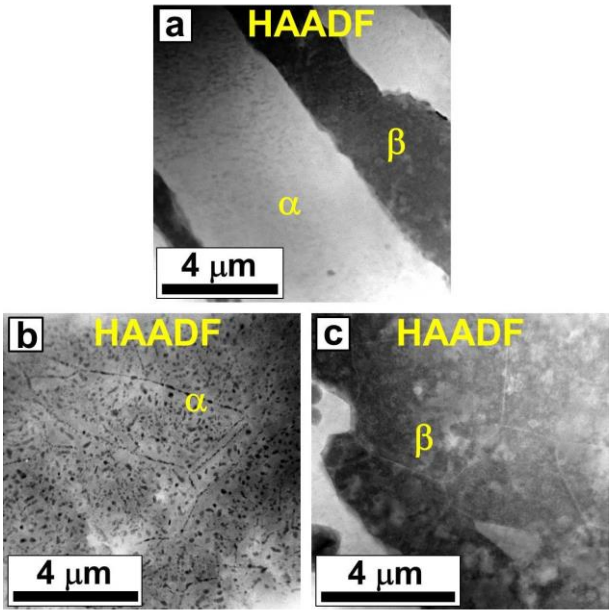

Figure S5. The average size of α and β phases are 14.7 mm, and Li-rich and Mg-rich clusters are formed in the α and β phase, respectively after extrusion. Microstructure of extruded Mg-Li alloy. (a–c) HAADF images showing the size of α and β phases and the distribution of Mg and Li atoms. Bright and dark contrasts correspond to the Mg-rich and Li-rich regions, respectively.
